# Supplementary material for: A qualitative exploration of mental health services provided in community pharmacies
Source: PLoS One. 2022 May 12;17(5):e0268259. doi: 10.1371/journal.pone.0268259 (PMC9098086; doi:10.1371/journal.pone.0268259)
Supplement: S1 Appendix — (DOCX) [file pone.0268259.s001.docx]

| S1. Factors moderating pharmacists and patients’ empowerment in mental health and supporting quotations resulting from the thematic analysis in Round One | | | |
| --- | --- | --- | --- |
| **External factors moderating patients' empowerment in community pharmacy** | | | |
| **Community pharmacy setting** | | | |
| **Accessibility** (Easily reached, entered, or used by patients, especially those who have a disability.) | | | *CPUn5: “We look into some things that you need to do with the accessibility of the pharmacy, and I think that applies to people with mobility struggles and I think a lot of that can overlap with mental health and how welcome people feel in the pharmacy.”*  *PSRn3: “At the pharmacy we can actually do things like Webster packs and things like that and see the treatment plan, things that allow more accessibility, but I think it still is a barrier because we don't necessarily see the patient as often.”* |
| **Physical layout of the pharmacy**. (Strategic arrangement of the community pharmacy space to enhance patients' privacy and the provision of services (e.g., consultation room) | | | *CPRt2: “The way the physical layout has been set up so that people feel comfortable with the privacy that they have with sharing their own personal information to keep things discreet”*  *CPUt4: “Physical layout of the pharmacy and the, I guess, relatively recent mandating of the private consulting room or area would be another important facilitator.”* |
| **Privacy** (Importance of patients' anonymity and confidential information.) | | | *CPRt1: "In a smaller town, if people know the work that is done in your pharmacy, they are not going to come in and want to talk to you about anything. I know everyone in there and they probably think that like their privacy might be breached."*  *PSUn4:"Privacy can be something that people do get concerned about, but if we're able to be respectful and professional, then you know they're going to come back and trust us."* |
| **Pharmacists and pharmacy staff** | | | |
| **Accessibility** (Easy access to pharmacists and pharmacy staff) | | | *CPUt5: “Accessibility, we're, you know, quite accessible to our community and our patients or clients, especially to those with mental health conditions they may come to us for another reason and then they will get into the other problem”*  *CPRt2: “We're really the first point of healthcare in the community where people can easily approach, besides the GPs and probably before getting a psychologist”* |
| **Approachability** (i.e., friendly, less formal). | | | *CPUn7:"You [pharmacist] can almost approach them [the patient] from a casual conversation, rather than the clinical conversation and that could help avoid the confrontation to speak about mental health"*  *CPRn3:"Pharmacists and assistants make it very conversational so they make it easy for the customer to, you know, have a chat with the pharmacist and it just that really they focus on caring, so it doesn't matter whether it's really small issue or a big issue."* |
| **Continuity of care and follow up**  (Familiarity and rapport with patients due to continuous interactions) | | | *PSRn2: “We're very isolated. Like a lot of people around us run stations and then kind of come to town like once a month, once every two months. So, you find that you don't have that kind of consistent contact with them, and they also don't have that consistent contact with other people. So, it may just be their family out on that station, and they don't have contact with anyone for months. A lot of them have their medications posted as well.”*  *PSRn3: “We see them every week and because we've got that good relationship, we can sort of be as a part of the pathway in referring them back or just supporting them.”*  *CPUn2: “I think pharmacists get to know their customers more as well. They can identify when somebody is not quite right. Maybe they [patients] do not seem like themselves, maybe something happened in their life and yeah, the pharmacist is more likely to be aware of that and be able to respond.”* |
| **Empathy and Reassurance** | | | *CPRt4: “It kind of gives them [the patient] a bit more reassurance that we're there to help out or like a bit of a safety net for any extra questions that they might have”*  *CPUt1: “We [the pharmacist] can kind of provide patients the reassurance that a lot of other people have similar issues and things like that, and it's not something to be embarrassed or ashamed about and kind of yeah just reassure them that there is support out there.".”* |
| **Provision of high-quality services according to patients needs** | | | *PSRn5: "Providing the correct sources constantly, not letting patients kind of walk out of the store without fully feeling they've gotten the best service they can."*  *CPUn7:"I think it's really also important to have that discussion with the patients and see what sort of support networks they've got in their own life already and making sure that they utilise those, whether it's friends or family or whether they have a good relationship with a local doctor or anybody else."* |
| **Additional services and resources available at the pharmacy** | | | *CPRn3:"We give patients information regarding the mental health online tools available. Showing them those sorts of things that they can do it is helpful as well."*  *CPUt5:"Yeah, we have the opportunity to add like nonpharmacological management in with our counselling as well, which patients can also benefit from"*  *PSUn2:"We, the pharmacy I work at, often do home delivery and so you can actually check on your local patients when you're there too."* |
| **Rapport and trust** | | | *CPUt2:"You have those regular customers who are trusting you with their life. You want to make sure that even if they're not regular, they can still trust you to give them the advice that they need."*  *CPRt5:"Rapport and trust. I think a lot of the time particularly in rural areas, the pharmacist tends to stay and put down roots, but the doctors they go in and out and there's a different GP flying in weekly, monthly. So, I think that's a huge enabler for us as pharmacists, you get to know these people on a very personal level."*  *CPUn7:"Particularly people have like certain ages or genders if they don't trust the pharmacist as professional then they're not going to share their problems."* |
| **Verbal and nonverbal communication.** | | | *PSUn4:"If you walk in the door and somebody is mean to you, you're not going to be like.. oh! I would love to discuss my mental health."*  *CPRN1:"Have those listening and reflecting little skills as well, so it's sort of part of the skill set and having that kindness as well."* |
| **Individual factors moderating patients’ empowerment** | | | |
| **Confidence** | | | *CPUt3:"Kind of lack of confidence, are my feelings or is my mental illness valid? It is so important to go talk to someone kind of feeling that it's not important."*  *PSUn2:"Ability of the patient to discuss their problem. So, we have some patients with Webster packs that have a carer come in. One time a patient didn't have their carer come in, and he didn't have the ability to cope with the stress of communicating."* |
| **Consumer characteristics** | | | *CPUn1:"In the metropolitan area that we are in, we do face like a lot of diverse ethnic groups, and there are language barriers involved where some people may not know how to communicate with you, and you may actually find you struggle to communicate with them. That also comes into cultural barriers."*  *PSRn4:"A lot of people in rural areas have this stoic farmer sort of personality, and it's really hard to break through that or break the ice to that in a short period of time. So, it takes probably time to build that relationship and sometimes it's important to have those quite frequent conversations with patients when they haven't had the ability to be vulnerable."* |
| **Health literacy and Knowledge** | | | *CPRt1:"I think sometimes we have a lot of interactions with people who don't really have much health literacy at all."*  *CPUn2:"Patient's health literacy is a big barrier and their understanding of their condition and medications as well. I think that also plays a big role in how we educate them and how they understand the condition they are suffering and how best for themselves to deal with the outside things, like, you know, environmental or factors outside of medications."* |
| **Stigma** | | | *PSUn3:"The stigmatisms that are still surrounding mental health acts as a barrier for people wanting to seek help."*  *CPRt5:"The side effects of certain medications like the stigma [of the patient] around that and their idea that the medication can sometimes make it worse, or they feel it can make it worse."*  *PSRn2:"A lot of people just by hearing the word anxiety or depression for them it's not normal, and they feel like they're going to be judged."* |
| **Willingness to change and spend time at the pharmacy** | | | *CPUn6:"We do play a big part in the long-term treatment of those conditions as well and I think the patient's willingness to change makes a big difference in that as well."*  *PSUn2:"Most people, you know, they're busy. They want to get the script and go out. They don't really want to stand around and talk about mental health or if the pharmacies are busy, they don't want to wait. Time is a big factor."* |
| **External factors moderating pharmacists' empowerment** | | | |
| **Education** | Training | | *CPUn3:"Pharmacists' lack of skills or education necessary to manage high care patients. For example, someone who might be at risk of suicide or this or that, our current education is obviously on the medication aspect but, through my experience in university at least, I didn't get that much extra training on how to have some of those conversations."*  *PSR1:"Mental health, you know, it's always can be tricky. The more training and the more experience, the better."*  *CPUt2:"I think we really need to educate pharmacists to obtain that self-confidence in providing mental health services to patients, and then, once I've got that confidence, then being able to actually promote that to the local health professionals, as well."* |
|  | Awareness of the resources available | | *CPUn3:"I guess it's not a part of I guess core knowledge, like it's an optional thing for pharmacists, although definitely seems important seems like everyone should be doing it but you're going to have like a large percentage of pharmacists that probably still don't have that training."*  *CPUt2:"After having done the course, I found that we should definitely all have been trained in it, but I wouldn't have known if I hadn't done it."* |
|  | Translation to practice | | *CPRt1: "I've done my mental health first aid training; however, applying it in practice within a reasonable amount of time, like you know, three to six months isn't necessarily very realistic."*  *CPUt3: "I think it's the practical application of the skill sets it's lacking. I think many pharmacists have a lot of knowledge, and we are really good at going and reading and finding out more but actually translating that into practice is quite difficult."* |
| **Availability, support and relationship with other healthcare professionals** | | | *CPRn2:"We have limited people [healthcare professionals] out here to see as well. If you're in Melbourne and Sydney like you've got more people to contact, more opportunities and different professionals, like psychiatrists and psychologists here are very short staffed."*  *PSUn4:"One of the big things I find is like the communication that the pharmacy assistants and the pharmacists have with the doctors because if there are any issues or if you know, people have questions, we can quite often, kind of get on to those doctors reasonably well."*  *PSRn3:"I don't abuse that relationship, and if I'm ringing for a referral, they tend to take it quite seriously. I guess developing professional relationships and collaboration between different disciplines as well."*  *CPUn2:"I know sometimes it can be so hard to actually get in contact with certain doctors even to get past their receptionist or secretary."*  *CPUt1:"Sometimes, it's not really the relationship that all pharmacists have. Unfortunately, I’ve had some pretty horrible experiences with doctors."* |
| **Pharmacists’ integration** (Referral pathways, standardised practice, and feedback loop) | | | *CPUn2: "There are all these services, but we don't know about them. Nobody talks to us about them. We don't always know, and even if we know about them, it's not like we have access to referring patients specifically to other health professionals."*  *PSUn4: "If the doctor or specialist want to increase the dose and tell the patient to take two of the same tablet when he comes into the pharmacy, there will be no documentation anywhere. We're just going on with the word of the patient."*  *CPRn4: “If I encounter someone who's really suffering from mental health illness, I'm really not sure if there is a document or guide that we can follow, where should I refer the patient? There’s no guide for us.”*  *CPUn6: “W*hat's lacking in fact in pharmacy, there's no history and people don't have the habit to document interactions.” |
| **Promotion** | | | *PSUn2:"If you don't advertise the services to patients or other health professionals, it's not going to work."*  *CPRn3:"I guess it's a barrier that not everybody knows that you can come to the pharmacist and talk about your mental health."*  *"I think we need that kind of like national campaign or awareness campaign on what pharmacists can provide then people know about that."* |
| **Role recognition** | | | *CPUt3:"We need to be more involved with the primary health networks and that's because they have extra funding for extra mental health services and, at the moment they don't recognize us, and that's because not many of us are on the Clinical Council of the primary health network."*  *CPUn1:"We haven't been included in the whole process and it gets a little bit disjointed sometimes".*  *PSR3:"I also don't think people recognize that we're in a place to actually help either, so some of my family members that have needed assistance haven't realised that my training actually somewhat covered understanding of disease states and facilitating those things."* |
| **Workplace culture and support available**  (Relationship, communication, and collaboration between employees.) | | | *CPUt7:"Healthy workplace culture when I'm talking about treating mental health for either staff or patients."*  *CPUn1:"The quality of service that you can consistently provide for mental health patients because sometimes, the pharmacy doesn't support that, they don't have enough resources, your employer doesn't support it. That's a major barrier."*  *CPRn4:"The relationship the staff have with each other, that reflects to the patient."* |
| **Resources** (Availability of assets that pharmacists and pharmacy staff can draw on to function effectively) | | Remuneration | *PSUn4:"At work, I know that financially my employer is probably not going to be the most responsive of letting me go to do the course during work time when money wise, it's not going to bring much to the business."*  *CPRn4:"Remuneration and the lack of funding for service provision around mental health services. So even if that's just a basic consult right up to something that requires more comprehensive review, there's no specific remuneration or funding model for them."*  *CPUt3:"You can't take a pharmacist off the floor if you are a busy pharmacy without some kind of remuneration. It doesn't make sense economically, and it is a business as well at the end of the day."* |
|  |  | Staff availability | *CPRn1:"Having two or three pharmacists to be able to provide the services."*  *CPUn2:"In the pharmacy that I work at, they've got more pharmacists on duty and more focus on professional services, so that's sort of facilitates, in my opinion, gives you a bit more time to spend talking to patients."*  *CPUt5:"When you don't have enough staff, then you're rushing other services you provide and that's going to be a risk."* |
|  |  | Time (Time availability and priority to provide mental health services) | *CPUn2: “Time. I know pharmacists are very often run off their feet with the number of things they have to do. I think having enough time to communicate effectively making patients feel understood is really important to their own mental health and how cared they feel.”*  *PSUn1: “It would be much better if we could have time away from the counter and to take the person into our consulting room and sit down and talk with them.”*  *CPRt4: “In Community pharmacy, one of the biggest issues is our lack of time.”* |
| **Individual factors moderating pharmacists’ empowerment** | | | |
| **Confidence** | | | *CPUn5:"A massive one that I find for service any service delivery, but especially mental health is the confidence of the pharmacist to deliver that that service."*  *PSRn2:"But sometimes it's hard to like, how do I start talking about it without upset anybody or make anybody have that trauma that they're potentially experiencing. It's like, how do we teach people to start the conversation."*  *CPU6n:"Us being young pharmacists, we don't have that confidence and kind of experience and starting those conversations."* |
| Skills and knowledge | | | *CPUn2:"Drug knowledge itself as pharmacists recognizing what they're talking about but also understanding why."*  *CPRn4:"You easily can know the side effects of any medications that will affect any mental health, so you're the one who's really going to give advice to these people."*  *CPUt2: “I think pharmacists play such a big role in, you know, communicating the risk-benefit of taking or not taking the medications and assessing patients’ adherence to their medication. Like we do play a big part in the long-term treatment of those conditions”* |
| **Mental toll**  (The effect that the provision of mental health services has on pharmacists and pharmacy staff.) | | | *CPUt5: “It takes a toll on pharmacy staff, and it does. I think not having some kind of support, you know, like an online I guess psychologist or something that we could do a break with at the end of the day,”*  *CPRn3: “Pharmacists’ own emotions, their own mental health is important as well.”* |
| **Pharmacists’ awareness of their role** (scope of practice). | | | *CPUn1: "I think it's important to understand where we fit in so that we're not duplicating efforts."*  *CPRt5:" It is important to understand where your practice fits in the wider context.”* |
| **Receptiveness and Willingness** | | | *CPUt1:"Some people might see it as wasting your time doing that [provide mental services] when you're getting nothing out of it in the end."*  *PSRn2:"It's easier to sort of almost not deal with it or pretend you're not qualified, so I feel that would be a barrier for a lot of pharmacists."*  *CPRt4:"It comes down to obviously time and the remuneration for our services because how much passion can you put into Mental health if there isn't something to back you there."* |
