# Supplementary material for: A qualitative exploration of mental health services provided in community pharmacies
Source: PLoS One. 2022 May 12;17(5):e0268259. doi: 10.1371/journal.pone.0268259 (PMC9098086; doi:10.1371/journal.pone.0268259)
Supplement: S2 Appendix — (DOCX) [file pone.0268259.s002.docx]

| S2. Factors highlighted for pharmacists’ empowerment in mental health and supporting quotations resulting from the thematic analysis in Round-Two | |
| --- | --- |
| **Governance** | *CPR5: “Having support for a structure or something to provide these services like, if you think about something like vaccination when we started vaccinating, we had guidelines that told us how to set up that service. So, there's kind of, I guess, some of that link to legislation in the States, but there's like organisational support for how you run that service to not having to think of it yourself.”*  *CPU3: “Guidelines come into play: mm hmm maybe clinical guidelines for what we are going to do.”*  *CPU1: “I think that guidelines play a huge role in defining what our scope of practice is and what the competency levels need to be. We have practice standards and guidelines for a lot of our professional services for that reason, I know that they're not used exactly how they're intended and there's work to improve some of those things, but they are what you're held accountable to if you are getting in trouble.”*  *CPR 2: “Maybe having an external sort of organisation and that had these kinds of pharmacists who can continually like help you become better.”*  *CPR3: “One of the most critical ones is the governance, so having proper steps that we can follow.”*  *CPU5: “The other key is obviously governance as well, so people need to know what to do and have the guidelines to sort of back their implementation of service.”*  *CPU6: “In terms of governance, you really need buying from the peak bodies to set some standards across the community pharmacy sector.”*  *CPR5: “I would probably say that there still needs to be some structural support in place to know what to do in the broad categories of presentations that you might get. Unrelated but half related to this, my research showed that pharmacists love being told what to do.”* |
| **Consistency and relationship with other healthcare professionals** (provision of high-quality services to patients to increase recognition and integration) | *CPR1: “I think one of the big priorities should be a consistency of the high-quality service which then will feed into a good relationship with the other healthcare providers and therefore the referral system, because the respect and referral from other healthcare professionals come from the trust that you're going to provide a high-quality service.”*  *PSU1: “I would probably just add to add weight to the quality assurance discussion, so I think that if we have a quality service that other health professionals can you know, rely on in terms of the feedback they get from pharmacists about patients and vice versa, then I think that would sort of perpetuate this idea of the pharmacist role and that would just empower individuals along the way.”*  *PSU2: “It's the same thing with pricing that kind of thing as well, like for sure some Discount chains out there, or someone is going to try and undercut the prices and then the quality [of the service] will go down as soon as it's undercut. You know, in order to make it successful you need to think about those things.”*  *CPU5:“ I think sometimes like people think there's a really big barrier but it's more that they’re under-resourced to actually use the power they have to do it, something like that it's like even if a pharmacy really wanted to, they could work with the primary health network and set up all of the local kind of things you need for mental health service to work well, the problem is you don't know whether your other colleagues are going to follow what you're doing, whether it's going to become a systemic thing, whether the quality will be like continued.”* |
| **Workplace culture and support available** | *CPR2: “When you work in a Community pharmacy and say you're the only pharmacist, you didn't get to eat lunch, you didn't get to go to the toilet for five hours and someone comes in and he's like help me with my depression and you're just like... if they [the pharmacist] are not supported by the workplace for that kind of thing, it's very likely they're going to reflect that on the patients. It's actually a major workforce problem, and I think that I would be worried about the quality of services sometimes because if the bosses are treating a pharmacist like that, it's likely that it will come out, you know what I mean yeah.”*  *CPU6: “One of the barriers or enablers or whatever way you look at it, it’s the way that that team operates in terms of the screening and referral of a patient who needs mental health kind of follow up so whether it's initially a pharmacy staff member that escalates their concerns to a pharmacist… I just didn't know whether we captured that team kind of collaboration.”*  *CPU3: “Quality control as well, so if you don't have a second pharmacist on, you can't vaccinate because it is not appropriate, and that removes some of the pressure off the pharmacist if you're alone.”*  *CPR4: “Pharmacies are really small businesses so when you start a new service, literally the pharmacist is reading this protocol and like trying to figure out what to do because no one has ever done this before; you've never seen anyone do it before, so you're trying to figure out how to make it work for your patient sake, and that implementation process on its own is sort of you don't get a lot of support for it.”* |
| **Pharmacists’ education and training** | *PSU1: “Pharmacists really we appreciate, and we'd like being better at what we're doing, so if you can give us a way to continually improve and that includes that kind of feedback loop, it actually would help with that empowerment. You wouldn't feel like you're alone doing it all the time, which we often do like someone gives us a random service and we're just supposed to become better and better.”*  *CPR2: “Yeah, I think education is right, right now, we know how to recognize it, but we don't know where to go from there and we may see a patient struggling, but we don't necessarily know what the next steps are.”*  *CPR4: “I think education and training of pharmacist is like the case starting point, so we know where to start, we know like how to recognize the services available things like that which then leads on to like building out confidence as well.”* *“Especially, being in rural areas, we don't have access to that kind of training, all the time…I’m from Broken Hill (NSW) and for me to do my Mental Health First Aid, we had to travel to do it in person, which was a three-day trip for us, so I suppose you need to consider the logistics as well…always a big problem.”* |
| **Resources-Remuneration, time, and staff availability** | *CPU4: “The main thing that really makes it very frustrating to deliver these services is the remuneration that we have for the pharmacist to really do these things. You need a pharmacist who doesn't have to dispense.” “We just need pharmacists that they are basically a counselling pharmacist and doing all the professional services, rather than having to do the physical stuff of putting stock away, dispensing and all that stuff.”*  *CPR3: “Yeah, we need to be properly remunerated and to ensure that the cost isn't past patient, which I think it's really essential. We need to make sure that if a service something like a mental health service is rolled out across the Community pharmacy that they are the people that are involved.”*  *CPU2: “If we've got time and money, then we can do training, we can put services in, we can do marketing campaigns for recognition. If we don't have any money, we can't do any of those things and continue to rely on the goodwill of the pharmacist.”*  *CPR1: “Time is a big issue, you can have the education and recognize patients who need assistance, but ultimately if you don't have staff availability, time and the remuneration for that service you provide, you know, everything else will fall by the wayside.”*  *CPU5: “Our mental health needs to be looked at as well, because you need to be supported, to be able to support other people in the Community and that's something I feel that's what's lacking in the Community pharmacy yeah”* |
