# Supplementary material for: A qualitative exploration of mental health services provided in community pharmacies
Source: PLoS One. 2022 May 12;17(5):e0268259. doi: 10.1371/journal.pone.0268259 (PMC9098086; doi:10.1371/journal.pone.0268259)
Supplement: Attachment — Submitted filename: Reponses to reviewers R1.docx [file pone.0268259.s003.docx]

| **Reviewer 1** | | | |
| --- | --- | --- | --- |
| **Manuscript section** | **Reviewer 1- Comments to authors** | **Authors Response** | **Change(s) in manuscript** |
| **Introduction** | The background provides context for this research; however, I think that it is a bit long and unfocused. Specifically, it covers mental health problems globally and in Australia and their negative impacts on the economic side, and how COVID-19 deteriorates mental health problems. In my opinion, the real meat of the introduction begins on Page.5 line 104 because this is the introduction of the roles of community pharmacies in delivering mental health care in Australia. I think that the authors should re-assess the material prior to this paragraph and determine which information is most germane to meet this study's primary objective. In addition, I think the authors should highlight the novel of this study. | We thank Reviewer #1 and agree with comments on the introduction; it is now much improved – the following changes directly reflect Reviewer #1 recommendations: The background section has been reviewed and modified. The information about mental health problems globally, their economic impact and the effect that the COVID-19 has had on mental health problems has been cut down. Additionally, more information regarding the effectiveness of pharmacist-led mental health interventions, availability of pharmacy mental health frameworks and reports, and the factors previously identified as affecting the provision of pharmacist-led interventions has been added. | **Background section paragraph 1-5.** |
| **Methods** | Major Comment 1: There is a lot of duplication of methodology between the text and figure 1. I would encourage the authorship team to streamline presentation of information. I would also encourage authors put data analysis section together if applicable. | We agree with the reviewer’s comment. The methods section and Figure 1 have been reviewed duplicate information has been removed. | **Methods section and Figure 1.** |
|  | Major Comment 2:  The authors state participants were recruited using purposive sampling of focus groups. Please elaborate in greater detail on how this was done, including 1) What are the inclusion and exclusion criteria for recruitments? 2) Do authors include chain pharmacies or independent pharmacies? 3)Do those pharmacies offer any specialty services (e.g., mental health services) before recruiting into the study? 4) Do those pharmacies have similar volume of prescription dispensed per day/week or have similar pharmacist/staff ratios? Elaboration on the above details will give readers greater insight an assurance that the integrity of data (and subsequent conclusion drawn from them) are defensible | We thank Reviewer #1 and agree with comments #2. The following changes directly reflect Reviewer #1 recommendations: Additional information regarding participant inclusion criteria has been added in the methods section. Moreover, further information about the characteristics of the participants demographics has been added to Table 2. | **1.Methods section-Participants’ selection and recruitment section**:  “Participants were recruited using purposive sampling. The following inclusion criteria were applied:  •Community pharmacists (pharmacy owners and employees) and pharmacy staff;  •working in any type of community pharmacies (e.g., chain, independent, banner);  •located in metropolitan, regional and rural areas of New South Wales (NSW).”  **2. Results section- Table 2. Participant demographics (Round One)** |
|  | Major Comment 3:  Line 158. The author state that “Each focus group meeting was divided into two stages” in Round one. However, only the first 4 focus group went through 2 stages based on the context and figure 1. Please clarify if there is a discrepancy existing between the statement and figure 1 or provide more information or purposes why choose to use different methodology in Round 1? In addition, consider adding the aim of the Round 1 | In order to clarify the information provided in the methods section further information regarding the data collection process in the two stages (i.e. December and January) of Round One has been provided. | **Methods section-Data collection:**  “The focus groups were facilitated by two researchers (JR, SDG). An additional researcher was an observer in the group discussion (CCG). A focus group guide (Fig 1) was developed prior to the commencement of the study which was based on previous literature. Data collection was conducted using two different techniques. In December of 2020, data were collected using a combination of the Nominal Group Technique and open discussion questions. In January of 2021, data were collected using open discussion questions.  **December of 2020**  Four focus group were conducted. Each focus group meeting was divided into two stages:…”  “… **January 2021**  The remaining two focus groups were conducted as an open discussion to obtain further insights of the information retrieved in the previous focus groups. The facilitator (JR) posed several questions to participants for discussion (Fig 1), developed based on the results obtained from focus groups 1-4.” |
|  | Major Comment 4: Line 242. The author state that “The goal was to recruit between 4 and 7 participants per focus group” in Round 2. The number of participants in Round 2 per group was less than Round 1 and the number of focus group was also less than Round 1. Would it be a potential bias in data reporting or validation? | Thank you for your comment. In Round One, we recruited 10 participants per group to account for potential nonattendance. However, according to the literature, large groups of 10 or more are difficult to control, limiting participants’ opportunities to share insights and observations. Moreover, group dynamics may also be affected when participants want to but are not able to describe their experiences. Despite this, invitations were sent to all participants from Round One and 16 participants were recruited in Round 2. Five of them dropped out because of work commitments before the commencement of the study. Therefore, the first focus group in Round Two included 6 participants and 5 participants in the second one as the previous focus group. This information has been added in the results section.  Additionally, the number of focus groups in Round two was not previously set and 2-4 focus groups were expected to be enough to validate the findings of Round One as there was not much variability within the ideas obtained in the previous six focus groups. This was confirmed through the overall agreement across participants in both Round 2 focus groups. | **Results section, Round Two-Validation of findings**  “Sixteen participants were recruited in Round Two. Five of them dropped out before the beginning of the focus groups due to work commitments. Eleven participants (i.e., community pharmacists and staff located in urban and rural/regional areas) were allocated to the two follow up focus groups to validate the findings from Round One. Participant demographics are provided in Table 5. The average duration of the two focus groups was 86 minutes (SD 17.6).” |
| **Results** | Major Comment 1: Author mentioned that there is a network of 5,822 pharmacies in Australia in the introduction section. Would the results of this study have any potential bias because of the sample size? | Participants in this study are community pharmacists’ and pharmacy staff located in areas of NSW. The number of participants in this study is in line with what is available in similar qualitative research. We are aware that the information retrieved from this particular group may differ from other community pharmacists and pharmacy staff in different situations. However, due to the diverse characteristics of the participants and the number of focus groups conducted, we believe that the results of this study provide a comprehensive view of the current situation in community pharmacies. Moreover, the goal of this qualitative study was not to generalize but rather to provide an in-depth understanding of community pharmacists and staff experiences with the delivery of professional pharmacy services through the study of these particular cases. Thus, we have not made a revision in response to this Reviewer query. | Not applicable (N/A) |
|  | Major Comment 2:  The authors identified 24 factors that enable or hinder mental health service delivery in community pharmacies. It is a little bit unclear how did those factors be identified? Were they identified through the 2 nominal questions in Round 1 only or combined with thematic analysis later? Consider elaborating in greater detail on how this was done. | Additional information has been added to the results section to clarify this reviewer #1 concern. | **Results section, Nominal group voting:**  “From the first four focus groups of Round One, twenty-four factors that enable or hinder mental health service delivery in community pharmacy were identified using the Nominal Group Technique (Tables 3 and 4).” |
|  | Major Comment 3:  What were the themes from Round 1 and Round 2? I would encourage the authors to use more tables to summarize the themes with supportive quotes from Round 1 and Round 2. It is good to have great details in the texts, but sometimes it is hard to follow, especially since this manuscript has two rounds of information with multiple themes. Therefore, I would recommend streamlining some texts and putting the additional quotes into tables if applicable. | The following changes directly reflect Reviewer #1 recommendations: We believe that these changes have made the presentation of the study much clearer for the reader: The themes and supporting quotations resulting from Round 1 and Round 2 have been included in Appendices S1 and S2, respectively. The information provided in the results section has been modified. | **Appendices S1 and S2:**  S1. Factors moderating pharmacists and consumers’ empowerment in mental health and supporting quotations resulting from the thematic analysis in Round One.  S2. Factors highlighted for pharmacists’ empowerment in mental health and supporting quotations resulting from the thematic analysis in Round-Two. |
| **Discussion** | Discussions: Major Comment 1: Paragraph 5. The authors discussed the impact of governance in this paragraph. Per table 5, governance is defined as the availability of resources to guide, support, maintain and improve the reliability, safety, and quality of the services and standardise mental health service delivery in community pharmacy. There is no clear relationship between governance and policy. Consider providing more details about the governance in the text and how it links to the policy in the discussion. | The following changes directly reflect Reviewer #1 recommendations In order to clarify the concept of governance and its relationship with policy, the definition provided in Table 6 has been modified, and additional details have been added to the discussion section. | **Table 6:**  Governance: “Having the set of processes, regulations, policies and resources to define, regulate and standardise mental health services delivery in community pharmacies to enhance safety, reliability, and quality of care.”  **Discussion, fifth paragraph:**  Good governance has also been associated with improvements in the safety and quality of health care services by implementing policies, educational materials and processes for improvement. It determines how health services are delivered and directly influences the safety and quality of services[60]. |
|  | Major Comment 2:  Limitation. The authors should state generalizability as a limitation due to the sample size. Generalizability may further limit the methodology which considers the experiences of participants which may vary considerably among different pharmacies. In addition, selection bias may be existing because some pharmacists were certified, and some were not. | We thank Reviewer #1 for these suggestions. However, we offer alternative views: due to the qualitative nature of the research, the goal of this study is not the generalisation of the findings to all community pharmacies. Instead, it seeks to provide some insights regarding the current situation in some community pharmacies from the perspectives and experiences of participants.  Regarding the participants’ characteristics, we considered that including certified and not certified pharmacists and pharmacy staff would allow us to explore the experiences of a more diverse population of pharmacists. Thus, the issue of mental health service delivery in community pharmacies has been explored from different perspectives. Moreover, as mental health service delivery in community pharmacies is still limited, a finding from this research was that there was consensus between participant’s opinions regardless of their situation. **The limitation section has been updated in response to this Reviewer #1 concern.** | **Discussion section, strengths and limitations:**  “The findings of this research may not represent the full spectrum of opinions/experiences of community pharmacists across Australia. Therefore, their applicability to community pharmacies located in other areas may be indicative but limited. However, the inclusion of participants with diverse characteristics (e.g., trained and not trained pharmacists) has facilitated exploration of the issue of mental health service delivery in community pharmacies from different perspectives.” |
| **Conclusion:** | Major Comment 1: In the method section, the authors did describe the plan to evaluate the role of pharmacies during COVID-19 pandemic. To me, it is a bit inappropriate to draw the conclusion because it was not derived from this work in the results section | We agree with the reviewer’s comment, and we have modified the conclusion accordingly. | The qualitative work undertaken in this study explored community pharmacists’ and pharmacy staff experiences and perspectives regarding the provision of mental health services in community pharmacy. |
| **Additional notes** | 1. Figures: I would recommend increasing the resolutions of all figures | We have ensured that Figure quality parameters meet the editorial requirements for PLoS-One | N/A |
|  | 2. Line 255. The author state that “participants were presented with a framework”. It is a little bit unclear how the framework was presented. Did participants see the figure 2? | Yes, figure 2 was included as part of the power point presentation showed to participants during the zoom meeting. This was stated in the original paper. | **Methods section, Round two: validation of findings, data collection:**  “Following the discussion, the framework (Fig 2) was presented to the participants as part of the PowerPoint presentation. Several questions related to the framework were asked to participants (Fig 1).” |
|  | 3. Table 1. The table shows that some pharmacists and pharmacy staff who were not Mental Health First Aid certified. Is this a nationwide or statewide level certification? Would the results be different if study only included pharmacists and pharmacy staffs who are Mental Health First Aid certified? | As stated above, data collected from pharmacists and pharmacy staff Mental Health First Aid certified did not differ from those not certified. This may be explained as the provision of mental health services in Australian community pharmacies is still quite limited due to external factors such as remuneration, lack of integration within primary care which affected participants regardless of receiving or not receiving training. |  |
|  | 4. Line 592. Missing figure# or word in the beginning? | The sentence has been modified. | **Results section, Thematic analysis:**  “Final proposed framework for pharmacists, pharmacy staff and consumers’ empowerment in mental health.” |
|  | 5. Consider to include what is the next step of the future research to expand the role of community pharmacy in mental health delivery in Australia | We agree with the reviewer’s comment and the conclusion section has been modified to include the next steps for future research. | **Conclusion section:**  “Future research should assess the applicability of the framework in practice in other settings. Furthermore, customer’s perspectives and experiences regarding mental health service delivery in community pharmacies should be explored to validate the results of this study and identify additional factors affecting their empowerment.” |
| **Reviewer 2** | | | |
| **Manuscript section** | **Reviewer 2- Comments to authors** | **Authors Response** | **Change(s) in manuscript** |
|  | This is a small study, and reflects a potential trend?...I would widen the population of community pharmacists to at least statistical significance, and then present the date as a potential alternative therapeutic modality which may supplant others......... The big error that I find is thst this only addresses prescription meds, and not OTC meds which are 'prescribed' by the pharmacists.......that of course is an established mode, but some are also of precriptive use if dose is doubled.....many are OTC at 1/2 dose.....hmmmmm... | Thank you for your comment. Statistical significance does not apply to qualitative research. Moreover, this study does not address any type of medicines as the objective is to explore experiences and perspectives of community pharmacists and pharmacy staff regarding mental health service delivery.  We found Reviewer #2 comments to be inconsistent with the views of the other two reviewers and our own views. We rebut the recommendations around quantitative analysis. Thus, we have not made any revisions in response to Reviewer #2 comments. | N/A |
| **Reviewer 3** | | | |
| **Manuscript section** | **Reviewer 3- Comments to authors** | **Authors Response** | **Change(s) in manuscript** |
|  | This paper describes a round of focus groups with pharmacists and pharmacy staff exploring facilitators and barriers to mental health services in community pharmacy. It addresses an important area of trying to progress much needed support for pharmacists’ roles in mental health care. It is generally well written but I have two key issues with the paper as present: • Length – although I note that the submission guidelines do not stipulate a word count, I feel this paper is excessively long and the paper could be significantly shortened, almost be half. The key messages get lost and a succinct paper is much clearer for the reader. I estimated the word count to be more than 8700 words. • The proposed framework suggests factors moderating patients’ (note comment below about language and use of term patient) self-empowerment. However, I have major concerns about this part of the framework as consumers/people with lived experience were not part of this research. Data was only gathered from pharmacists/pharmacy staff and I feel it is too much of a reach to then propose what moderates consumers self-empowerment based on the data. I suggest framework be revised to acknowledge this and identify consumer involvement in this area as where further research is needed | We thank Reviewer #3 for the view that the paper “addresses an important area of trying to progress much needed support for pharmacists’ roles in mental health care.”  In response to recommendations on the length of the paper, revisions have addressed similar comments from Reviewer #1 (addressed above). Consistent with recommendations from Reviewers #1 and #3:   - The manuscript has been reviewed in its entirety and has been reduced in length. - We agree with the reviewer’s comment, with respect to the framework and have revised the discussion of limitations section and included this issue as a limitation of the paper. | **Discussion section, Strengths and limitations:**  “A further limitation of this study is that the framework proposed includes factors moderating consumers' empowerment in mental health from pharmacists’ and staff perspectives. Therefore, this should be considered as some of the factors identified may differ from future research including customers' perspectives.” |
| **Background** | • It is currently very long and could be significantly cut down. For example, the first 1.5 pages could be condensed into 1 paragraph about the prevalence and burden of mental health problems | In response to recommendations on the length of the paper, specifically with reference to the Introduction section, revisions have addressed similar comments from Reviewer #1 (addressed above). Consistent with recommendations from Reviewers #1 and #3: | **Introduction section:**  “The burden of mental health problems continues to grow worldwide with a 13% rise in mental health conditions and substance use disorders in the last decade [1]. The cost to manage many mental health conditions effectively is relatively low [1], however, there is still a substantial gap between people needing care and those who have access to it [2]. Poor mental health is estimated to cost the global economy around $6 trillion per year by 2030 due to poor health and reduced productivity [3].  In Australia, mental illness is widespread and has substantial impact at the personal, social and economic levels [4]. Four million Australians had a mental or behavioural condition between 2014 and 2015, a figure which increased to 4.8 million in 2017 [5]. It is predicted that almost half of the Australian population will suffer from a mental health problem at some stage in their lifetime [6]. Due to geographic and social isolation, people living in remote and rural areas in Australia have a higher risk of experiencing poor mental health and wellbeing, evidenced by the significantly higher suicide rates compared to metropolitan areas [7, 8]. Importantly, 4.3 million people were supplied with mental health-related medicine in Australia in 2018-2019 [9]. In 2019–2020, $1.4 billion was spent by the Australian Government on benefits for Medicare-subsidised mental health-specific services and $566 million on subsidised mental health-related prescriptions under the Pharmaceutical Benefits Scheme [10].” |
|  | • A variety of references are used for these figures on prevalence/burden. Ensure the primary source is being cited – e.g. Black Dog Institute flyer rather than data from the National survey of mental health and wellbeing | References have been reviewed and primary sources cited. | Per References section. |
|  | • Introduction to pharmacists’ roles in mental health describes what pharmacists can do, but it should summarise the evidence supporting these roles. In particular, what is known in the literature about barriers and facilitators to pharmacists’ roles in mental health care already? What governance/policy frameworks already exist for the pharmacy profession in the mental health space? In addition, what is known about barriers and facilitators to pharmacists’ roles more broadly in other areas that could be adapted here? The introduction is very long, but it could be much more succinct as well as better at identifying the gaps in the literature and how this study aims to fill those gaps. | The introduction section has been reviewed and additional information regarding the current situation and gaps in the literature has been added. | **Introduction section:**  “In recent years, key pharmacy bodies worldwide have published reports and frameworks highlighting pharmacists’ potential roles in mental healthcare. For example, the International Pharmaceutical Federation published a report showing how pharmacists and pharmaceutical organisations positively influence mental health care [27]. The National Health Service (NHS) of England also developed a framework of core mental health competencies for pharmacists [28]. A report published by the United Kingdom’s Royal Pharmaceutical Society [29] and a framework by the Pharmaceutical Society of Australia [30] articulate the role of community pharmacists as partners in multidisciplinary teams to support people with mental health problems. These reports also highlighted the barriers to pharmacists becoming partners in mental health care, such as lack of integration, recognition, time and confidence, which aligns with the available literature focused on other professional pharmacy services [31, 32]. However, there is a lack of research focused on community pharmacists’ perspectives and experiences regarding mental health service delivery in community pharmacies. Considering the perspectives and experiences of stakeholders involved in mental health service provision is essential to understand and identify factors moderating (i.e., facilitating or hindering) the provision of services within a specific setting [33]. Thus, the present study aims to examine the factors that support delivery of mental health services in Australian community pharmacies and proposes ideas for improvement, particularly in regional and rural regions.” |
| **Methods** | • I feel the methods a little confusing to follow. Round one has stage one and stage two with 4 focus groups using NGT, then a further two focus groups. These seems like two separate phases, and it seems like three rounds of focus groups not two? | The following changes directly reflect recommendations that were consistent between Reviewer #1 and Reviewer #3.: We believe that these changes have made the presentation of the study much clearer for the reader: Round One was divided between December of 2020 and January of 2021.In December 2020 four focus group using the NGT were conducted and in January two additional focus group were conducted as open discussion to further explore the information retrieved from the previous focus groups. The aim of these six-focus group was to collect information regarding community pharmacists and pharmacy staff experiences and perspectives about the provision of mental health services in community pharmacies. The methods section has been modified to clarify the data collection and analysis process. | **Methods section. Per changes made according to Reviewer #1 above** |
|  | • Page 9 line 224 – This sentence is a bit confusing and could be clarified? ‘to identify the underpinning reasons for participants selection and examine the data obtained’ | The sentence has been modified. | **Methods section, Round One, Thematic analysis**  “Thematic analysis of the six focus groups of Round One was conducted by two researchers (JR, CCG) and discussed with the research team (RAH, SDG) to justify participants’ ideas obtained during the NGT process and identify additional information resulting from the open discussions.” |
|  | • Page 10 line 244 – interview guide or focus group guide? | Focus group guide, the sentence has been amended. | **Methods section, Round Two, Data collection:**  “A focus group guide (Fig 1) and a Framework (Fig 2) were developed based on the results of Round One.” |
| **Results** | • ‘allocated according to their characteristics into six groups’ – explain how allocation occurred? And clarify did this mean that participants with training or no training were grouped together into focus groups? | The criteria to allocate participants in each focus group has been added to the results section. The presentation of this section is now much improved | **Results section, Participants’ characteristics:**  “A total of 40 participants were recruited in Round One. Seven participants dropped out prior to the commencement of the focus groups due to work commitments. The final 33 participants were allocated according to their role in the community pharmacy (i.e., pharmacists or pharmacy staff); the location of the pharmacy (i.e., urban, regional, rural or remote areas) and their certification status (i.e., trained in Mental Health First Aid or not trained) into six groups (Table 1). “ |
|  | • Table 1 – acronyms used here and in test of results. These are confusing to follow as reader needs to keep going back to see what they mean. I question whether they are useful? | No changes to the manuscript have been made in response to this comment. We believe that the acronyms are useful and effectively provide concise presentation of the information. |  |
|  | • Table 2 – some of the figures don’t add up. E.g. pharmacy staff – 6 born in Aust and 2 born in England but only 7 in total? For country of birth – if only 1 or 2 in non-Aust born category perhaps merge? | Table 2 has been reviewed and updated. | **Table 2. Participant demographics (Round One)** |
|  | • How do demographics compare to pharmacist population across Australia? Seems to be very high number of females and percentage born in Australia which may not be generalizable with pharmacy population. | According to the Pharmacy Board of Australia, the number of registered female pharmacists in New South Wales in September of 2021 was 6.163, and the number of registered male pharmacists was 3.783.^1^ This may explain the higher number of females in this study. Furthermore, the generalisability of findings is not a goal of this study due to the qualitative nature of the research.  ^1^Pharmacy-Board---Report---Registration-Data-Table---30-September-2021.PDF | N/A |
|  | • Page 17 – line 307 onwards. Thematic analysis referred to here – can it be clarified where this data comes from? I think it is stage two of round one? But it is confusing to follow | The following changes directly reflect Reviewer #3 recommendations The thematic analysis was conducted to the six focus groups of Round One. This has been clarified in the method and results sections of the manuscript. | **Methods section, Round One, data analysis:**  “Thematic analysis of the six focus groups in Round One was conducted by two researchers (JR, CCG) and discussed with the research team (RAH, SDG) to justify participants’ ideas obtained during the NGT process and identify additional information resulting from the open discussions [42].”  **Results section, Round One, Thematic analysis:**  “The referral pathways used by community pharmacists and pharmacy staff in urban and regional/rural areas have been identified due to the thematic analysis conducted to the data obtained in the six-focus groups of Round One.”  **Results section, Initial proposed framework for pharmacist, pharmacy staff and consumer empowerment in mental health:**  “Thematic analysis of the data obtained in the six focus groups of Round-One to further explore factors moderating mental health service delivery was conducted.” |
|  | • Should be consistent with describing demographics of participants in round one and two. Round two, readers only told that all are female? Perhaps merge these sections to the start of results to clarify number and demographics of all participants in each round – then go on to describe results? | The following changes directly reflect Reviewer #3 recommendations The demographics of the participants of Round Two has been included in Table 5. | **Table 5.** |
|  | • Page 22 – first sentence. Clarify grammar | The following changes directly reflect Reviewer #3 recommendations The sentence has been reviewed and modified. | **Results section, Round Two: Validation of results, Thematic analysis:**  “Most participants agreed that there is a need to empower community pharmacists to promote their role as mental health providers for example, through continuous training” |
|  | • General comment re quotes in results section. I suggest cutting down the number and length of quotes. A number of quotes are duplicative and others are very long which could be cut down to just present key points. E.g. quotes of page 24 could be cut down to one on guidelines and one on governance but currently there are 8 quotes. There are numerous examples of this that could help cut down the length of this paper. | The results section has been modified according to the reviewer suggestions. | **Results section.** |
|  | • I don’t think it is appropriate to name pharmacy chains – deidentify these. Also, these are not relevant to an international audience. | The name of the pharmacy chain has been removed from the supporting quotes. | Per the recommendation. |
|  | • Page 26 – line 528 – grammar | The sentence has been modified. | **Results section, Round Two, Thematic analysis-last paragraph:**  “The availability of remuneration and time for providing these services were highlighted as one of the most significant factors by participants in both groups.” |
|  | • Add footnotes to clarify any Australian relevant things in quote which need context for an international readership – e.g. medicare item number on page 28 | As per journal guidelines footnotes are not allowed. Definitions of Australian specific things have been added within the text. | N/A |
|  | • Table 5 – using wheelchair example of accessibility doesn’t seem to fit with what is often meant by accessibility here? Ie being able to talk to a pharmacist 7 days a week, without an appointment etc? | Accessibility has been added to the framework from two perspectives:   - Structural accessibility referring to the availability of resources in the community pharmacy setting.      - Community pharmacists’ accessibility alluding to pharmacists’ availability without appointment. | N/A |
| **Discussion** | • Both the introduction and discussion mention the COVID-19 pandemic, but this does not seem to be mentioned in any of the results? Particularly as this is how the discussion starts? While the literature describes the additional pressures placed on communities as well as pharmacists in the mental health space, the results here don’t describe this. I would suggest removing discussion relating to the pandemic, except perhaps to highlight the even greater role pharmacies have had to play because of the pandemic | The following changes directly reflect Reviewer #3 recommendations The text mentioning the COVID-19 pandemic has been removed from the introduction and discussion sections as it is not the paper's focus. | **Introduction and discussion sections.** |
|  | • The discussion is generally well written but there is significantly more literature known in the area of the effectiveness of pharmacist led mental health services – even systematic reviews that should be cited/discussed to give context of what is known in the area and how these results add value. As well as literature on barriers/facilitators to mental health service delivery. | The discussion section has been reviewed and additional information regarding effectiveness of pharmacists’ intervention in mental health care has been added. | **Discussion section, third paragraph:**  “Pharmacists' specific skills and drug knowledge was highlighted key facilitators in providing quality mental health care. The positive clinical impact of pharmacists' role in mental health has been previously demonstrated in the literature [46, 47]. Pharmacists' specific skills and drug knowledge was highlighted as key facilitators in providing quality mental health care. The positive clinical impact of pharmacists' role in mental health has been previously demonstrated in the literature [48]. Consequently, another study found that pharmacist-led mental health adherence interventions for patients with type 2 diabetes significantly improved psychotropic medication adherence in adult patients [49]. “ |
|  | • In addition, literature outside the pharmacy space should be discussed/compared to look at what is known that could contribute here. E.g. in the area of mental health stigma, patient empowerment. Do consumers even want pharmacists to have a role in mental health service delivery? | The following changes directly reflect Reviewer #3 recommendations Information regarding stigma and consumers’ willingness to receive mental health services has been included in the discussion section. | **Discussion section, fourth and fifth paragraph:**  “Consumer willingness to receive a mental health service was identified as a facilitator of mental health service delivery. In a study evaluating consumers' experience in a community pharmacy mental health program, participants recognised community pharmacists' positive influence on their mental health and well-being [50]. However, as reported in this study, the stigma of people living with a mental health issue discourages individuals from getting proper mental health treatment. These results are consistent with literature indicating that the mental health stigma of consumers and mental health services providers has been shown to be a barrier to the effective management of mental health [22, 51, 52].  Training and education in mental health were also reported as facilitators for service provision. Specifically, the importance of training to address stigmatising beliefs and stereotypes has been highlighted [53]. Indeed, mental health training has been shown to impact pharmacists’ confidence positively [54, 55]. Particularly in a study participants indicated comfortability discussing mental illness with community pharmacists trained in MHFA, revealing an opportunity for pharmacists to expand access to mental health services by being trained in MHFA and counselling about mental illness [56]. Thus, upskilling the pharmacy workforce in mental health should be prioritised.” |
|  | • Page 37 – MHFA – give reference and definition of what this is as not necessarily known to all. Or when mentioning a service such as MedsCheck – add footnote or a reference. | The definition of MHFA has been included in Table 1 as is the first time that appears within the text. | **Table 1:**  “Mental Health First Aid: early-intervention courses that increase mental health literacy and teach the practical skills needed to support someone experiencing a mental health problem, experiencing a worsening of an existing mental health problem or in a mental health crisis until appropriate professional help is received or the crisis resolves [44].” |
|  | • Discussion around MHFA. There is a pharmacy specific version of MHFA available which has pharmacy content and case studies. There is also a refresher course available already where participants can get re-accredited after a 3-year period. There is also significant literature in the pharmacy space about MHFA evaluation, the use of simulation to test MHFA skills and confidence. These should be discussed here. | MHFA training has been discussed. | **Discussion section, paragraphs 12-13:**  “Participants suggested ideas to improve the delivery of services by community pharmacists in mental health. Regardless of having undertaken the Mental Health First Aid course, participants commented on the possibility of including additional training with more practical scenarios relevant to community pharmacy and using people with lived experience. A Blended version of the MHFA course specifically tailored for pharmacists and pharmacy staff is currently available. This version consists of self-paced online learning modules followed by a practical classroom-based (face-to-face or live webinar) session using case-studies, videos and resources tailored to their learning needs [70]. However, the standard face-to-face MHFA course seems to be still the most common version used by pharmacists [71]. Pharmacists' [72] and pharmacy students’ skills after receiving MHFA training have been assessed in the literature (e.g., by using simulated patient scenarios) [73] and have demonstrated the positive influence of training on pharmacists' knowledge, attitudes, and confidence [55, 72, 73] . The use of real-life scenarios and the inclusion of multidisciplinary teams during training have shown improvements in the skills of healthcare professionals [74]. In the same way, studies including role-play as part of the training have demonstrated improved professional skills (e.g., recognition of possible scenarios and solutions, increased confidence and communication skills, promoted effective discussion, and active participation) [75, 76]. Despite this, some studies assessing pharmacists' confidence to provide mental health services using self-assessment tools have identified that participants often overestimate their confidence to deliver the services in practice after training [77, 78]. Nonetheless, including people with life experiences as part of the training to simulate real life situations should be considered to enhance pharmacists' mental health skills in practice.” |
|  | Limitations – I suggest adding a limitation about the lack of consumer involvement in this study. | The limitation section has been modified according to the reviewer suggestion. | “A limitation of this study is that the framework proposed includes factors moderating consumers' empowerment in mental health from pharmacists and staff perspectives. Therefore, this should be considered as some of the factors identified may differ from future research including customers' perspectives.” |
| **General:** | Language in mental illness. A few terms used in the paper can be problematic or stigmatizing when describing people living with a mental illness. People often do not like to be considered a ‘patient’ and you may consider an alternative term, e.g. a person with lived experienced of/or living with a mental illness, or mental health consumer. Also, the term ‘suffer’ has negative connotations and it is preferred to describe as ‘living with’ or ‘experiencing’ | We agree with the reviewer’s comment. The whole manuscript has been reviewed and updated according to the suggestion. |  |
