# Supplementary material for: A qualitative exploration of mental health services provided in community pharmacies
Source: PLoS One. 2022 May 12;17(5):e0268259. doi: 10.1371/journal.pone.0268259 (PMC9098086; doi:10.1371/journal.pone.0268259)
Supplement: Attachment — Submitted filename: PLOS ONE-reponses to reviewers R2.docx [file pone.0268259.s004.docx]

| **Reviewer 1** | | | |
| --- | --- | --- | --- |
| **Manuscript section** | **Reviewer 1- Comments to authors** | **Authors Response** | **Change(s) in manuscript** |
| **Introduction** | Consider removing the first paragraph and start with the current second paragraph to help readers knowing this is a study conducted in Australia sooner.  Consider combining the third and fourth paragraph together (or remove the fourth paragraph) because the fourth paragraph does not provide new essential information to readers after reading the previous paragraph | Thank you for your comment. The first paragraph has been removed. Additionally, the third and fourth paragraph have been combined according to the reviewer’s suggestion. | **Introduction section:**  “The burden of mental health problems continues to grow worldwide with a 13% rise in mental health conditions and substance use disorders in the last decade [1]. In Australia, mental illness is widespread and has substantial impact at the personal, social and economic levels [2]. Four million Australians had a mental or behavioural condition between 2014 and 2015, a figure which increased to 4.8 million in 2017 [3]. It is predicted that almost half of the Australian population will suffer from a mental health problem at some stage in their lifetime [4]. Due to geographic and social isolation, people living in remote and rural areas in Australia have a higher risk of experiencing poor mental health and wellbeing, evidenced by the significantly higher suicide rates compared to metropolitan areas [5, 6]. Importantly, 4.3 million people were supplied with mental health-related medicine in Australia in 2018-2019 [7]. In 2019–2020, $1.4 billion was spent by the Australian Government on benefits for Medicare-subsidised mental health-specific services and $566 million on subsidised mental health-related prescriptions under the Pharmaceutical Benefits Scheme [8].  Primary health care teams are playing a crucial role in the detection, management, and provision of long-term support to people living with a mental illness [9, 10]. The integration of community pharmacists as part of these teams has been supported and encouraged by government agencies and international institutions [11]. In Australia, community pharmacists are often the most accessible healthcare providers with a network of approximately 5,822 pharmacies [12]. Their accessibility has been evident throughout the COVID-19 pandemic, as community pharmacies have remained open providing essential services and access to medicines and health care. This is particularly important in regional and rural communities where timely access to other healthcare professionals and services may be limited. Specifically in 2004, the WHO recognised the importance of as community pharmacists as part of multidisciplinary teams [13] to optimise care for people living with mental illness, and their families [14]. Community pharmacists’ role in mental health includes, but is not limited to, the provision of information about conditions and psychotropic drugs, optimising treatment outcomes and quality use of medicines, medication adherence support, screening and early identification, provision of resources, referral to general practice or other services (e.g., hospital emergency services, drug and alcohol rehabilitation facilities) [15]. There is increasing evidence of the positive impact of pharmacists-led interventions have on adherence [16, 17], depression screening [18-21] and medication reviews for community-based mental healthcare consumers [22]. Nevertheless, the research in this area is limited and further studies demonstrating the effectiveness and cost-effectiveness of pharmacists-led interventions in mental health is needed.” |
|  | Line 82. A typo. C should be lower case.  Line 103. Remove “community”  Line 109. Consider adding New South Wales (NSW) here so that readers would know this is not a nationwide qualitative study. | Thank you for your comments. We have amended the manuscript accordingly. | **Introduction section:**  “In Australia, community pharmacists are often the most accessible healthcare providers with a network of approximately 5,822 pharmacies [12].”  “However, there is a lack of research focused on pharmacists’ perspectives and experiences regarding mental health service delivery in community pharmacies.”  “Thus, the present study aims to examine the factors that support delivery of mental health services in Australian community pharmacies in New South Wales (NSW) and proposes ideas for improvement, particularly in regional and rural regions.” |
| **Methods** | Line 120. Consider adding New South Wales (NSW) here so that readers would know this is not a nationwide qualitative study. | We agree with the reviewer’s comment. The information has been added. | **Methods section, Round One:**  “The aim of Round One was to derive information regarding the experiences and perspectives of community pharmacists and pharmacy staff regarding the delivery of mental health services in Australian community pharmacies in NSW.” |
| **Table 3 and table 4** | Not clear what does “Facilitator identified in urban and regional/rural areas” mean here. Do authors go back, and check where did each participant from?  Not clear what does “Barrier identified in urban and regional/rural areas” mean here. Like the comment for Table 3. | Thank you for your comment. As participants were allocated according to their characteristics (i.e., role in the community pharmacy; the location of the pharmacy and their certification status.), after conducting the Nominal Group Technique we were able to identify if the factor (i.e., barrier or facilitator) was mentioned by participants in urban or rural areas or in both areas. The text in both tables has been modified to improve clarity. | **Table 3 and table 4:**  “Participants located in urban or regional/remote areas.” |
| **Additional note:** | Figures: I would recommend increasing the resolutions of all figures. They are still not very clear. | Thank you for your comment. We have reviewed all the figures to ensure they have good resolution and are formatted according to the editorial requirements for PLoS-One. | Figures 1-3 |
| **Reviewer 3** | | | |
| **Manuscript section** | **Reviewer 3- Comments to authors** | **Authors Response** | **Change(s) in manuscript** |
| **Grammar –** | Still see patient used instead of consumer or person a few times (e.g. in abstract). Authors to check and ensure consistency. | Thank you for your comment. The manuscript and abstract have been reviewed and modified accordingly. | **Abstract and Main Manuscript.** |
|  | on page 61 line 189. Is ‘December of 2020’ – just ‘December 2020’ | Thank you for your comment. The text has been modified according to the reviewer suggestion. | **Methods section, Data collection.** |
|  | Page 61 line 193 – after text removed – sentence is very brief? | We agree with the reviewer’s comment. Additional information has been added to the sentence. | **Methods section, Stage 1: Nominal Group Technique.**  “The Nominal Group Technique (NGT) was applied to explore community pharmacists and pharmacy staff experiences and perspectives [32].” |
|  | Ensure acronyms spelled out first in text – ie MHFA. It is spelled in table 1 but not in text | Thank you for your comment. The MHFA is now spelled out in the text. | **Discussion section:**  “Particularly in a study participants indicated comfortability discussing mental illness with community pharmacists trained in Mental Health First Aid (MHFA), revealing an opportunity for pharmacists to expand access to mental health services by being trained in MHFA and counselling about mental illness [52].” |
| **Methods** | FG questions now in figure. This is better but perhaps text can summarise what they are about – e.g., on page 63 line 239-242 as this paragraph doesn’t say point of | Thank you for your comment. Further information has been added to the methods section. | **Methods section:**  “Two nominal questions were asked to participants to explore factors (i.e., barriers and facilitators) of mental health service delivery in community pharmacies (Fig 1).”  “ The facilitator (JR) posed several questions regarding their role as mental health providers in order to extend the knowledge that arose from focus groups 1-4 (Fig 1).” |
| **Additional comments** | There are now a lot of tables. Participant demographics from round one and two could be merged? I.e. with additional columns | We agree with the reviewer’s comment. Table 2 and Table 5 have been merged. | **Table 2. Participants’ demographics (Rounds One and Two)** |
|  | I couldn't see figures attached in revision. I assume these remain unchanged from version 1 | Thank you for your comment. Figures 1 and 2 were attached in the previous revision submitted to the journal. | **No changes required** |
